# Supplementary material for: rfaRm: An R client-side interface to facilitate the analysis of the Rfam database of RNA families
Source: PLoS One. 2021 Jan 15;16(1):e0245280. doi: 10.1371/journal.pone.0245280 (PMC7810343; doi:10.1371/journal.pone.0245280)
Supplement: Attachment — Submitted filename: Response to Reviewers.docx [file pone.0245280.s001.docx]

Editors

*PLOS ONE*

December 20, 2020-12-22

Dear Dr. Chen,

We thank you and the reviewer for reading our manuscript and providing constructive comments, as well as for giving us the opportunity to improve the manuscript and resubmit it. We believe we have now addressed all comments raised by the reviewer and you. These included providing additional examples and a description of each function of the package. We have now added a section describing the purpose, arguments and giving an additional example of usage for each of the functions in the package. Furthermore, other examples are available in the manual and vignette of the package.

We have also modified the formatting of the manuscript to match PLOS ONE´s style requirements as requested, and replaced the figure files by high-resolution figures in tiff format.

We thank you for your consideration of this resubmission. We appreciate your time and look forward to your response.

Sincerely,

Lara Sellés Vidal, Ph.D. (corresponding author)

lara.selles12@imperial.ac.uk
